# Perceived Effects of Agri-Environmental Management Practices on Public Good Delivery

**DOI:** 10.1007/s00267-026-02485-2

**Published:** 2026-06-29

**Authors:** Kina S. Harmanny, Franziska Komossa, Peter H. Verburg, Catharina J. E. Schulp

**Affiliations:** 1 Environmental Geography Group, Institute for Environmental Studies (IVM), Vrije Universiteit Amsterdam, Amsterdam, the Netherlands; 2 Department of Environmental Sciences, Land Use Planning Group, Wageningen University and Research, Wageningen, The Netherlands

**Keywords:** Biodiversity, Sustainable land management, Public goods, Ecosystem services, Farm management practices, Synergies and trade offs

## Abstract

European agricultural landscapes have historically been rich in natural features, such as hedgerows, herb-rich grasslands, and pastures, which provide essential public goods (PGs) such as biodiversity, climate regulation, and cultural landscapes. These multifunctional landscapes illustrate that agriculture is closely linked with natural resources, highlighting farmers’ dual role in both food production and the provision of environmental services. This study aims to expand our understanding of how different agri-environmental management practices affect PG delivery by surveying farmers in the Netherlands on their experiences and perceptions of implementing such practices. The 163 responses reflect a broad diversity of implemented agricultural practices with half of the respondents using 3–6 practices, while a small portion (10 farmers) incorporate all 11 targeted measures. Flower strips were the most widely adopted practice (60%) and showed positive impacts on biodiversity, landscape, and recreation. Additionally, we observed synergistic pairings, such as combining reduced pesticide use with flower strips. However, the results also highlight discrepancies between farmers’ perceived effects and existing literature, alongside notable challenges in implementation, such as the interdependence of neighboring farms. Farmers also noted negative spillovers, including yield reductions and increased costs. These findings suggest that effective PG delivery may benefit from more integrated management approaches and policy designs supporting bundled practices, aligning farmers’ efforts with broader environmental objectives.

## Introduction

Traditionally, European agricultural landscapes were characterized by features such as hedge rows, herb-rich grassland and cattle grazing in pastures. These features reflected the dependence of agricultural systems on natural resources, but also provided numerous agri-environmental-climate public goods (PGs) (Kratschmer et al., 2024) - that is, ‘’goods and services that are beneficial to the public and are thus highly desired by society but not readily traded on the market due to their inherent characteristics of non-excludability or non-rivalry” (Dwyer et al., 2015; Kratschmer et al., 2024). Examples of such PGs include e.g., clean water, biodiversity and soil functionality. These benefits are closely linked to the concept of ecosystem services and nature contributions to people which are defined as the benefits people obtain from ecosystems, which directly or indirectly support human well-being (Millennium Ecosystem Assessment, 2005; IPBES 2019). Traditional farm management thus made agricultural landscapes inherently multifunctional: ‘the simultaneous consideration of various positive (public goods) and negative (externalities) effects of agriculture’ (OECD, 2001). The concept of multifunctionality acknowledges that agricultural endeavors extend beyond their primary function of food production as these activities also play a role in shaping the landscape and offering environmental benefits (OECD, 2001; Song et al., 2020). However, post-world war II developments, primarily driven by the goals of ensuring food production and a stable income for farmers, led to a shift towards more intensive farming practices in Europe (Billen et al., 2021). The Netherlands was no exception. Drastic changes such as land consolidation resulted in a disconnection between nature, landscape and agriculture (Kuindersma et al., 2018) as agricultural production and landscape management no longer automatically coincided. These developments of scale enlargement and intensification resulted in environmental degradation, encompassing issues such as biodiversity loss, nitrogen deposition, declining water quality and soil degradation (Ellis, 2021). This contributes not only to surpassing global planetary boundaries (Campbell et al., 2017; Rockström et al., 2023) but also leads to the loss of PGs essential for local populations. Hence a broad consensus exists that agriculture must transition to a more sustainable path, emphasizing or regaining multifunctionality (Ramankutty and Coomes, 2016).

In practice, multifunctional sustainable land use can be achieved in different ways. First, it can be found in land management activities necessary for agricultural activity while simultaneously providing PGs (Power, 2010). In this case, management practices have additional effects on PGs not specifically targeted by the practice. For instance, farmers who stimulate grazing of their livestock not only enhance animal welfare (Cooke et al., 2023; Wilkinson et al., 2020), but also contribute to the visual appeal of the landscape (Van Zanten et al., 2016). In addition to these ‘unintentional’ contributions to PG delivery, farmers can deliberately alter their management practices to increase the delivery of specific PGs, for example, by altering mowing practices to protect farmland biodiversity (Johansen et al., 2019).

Agricultural policies emphasize supporting a wide range of practices, not necessarily coherent, with the aim to secure delivery of specific PGs. This emphasis is evident in the National Strategic Plans regarding the implementation of eco-schemes across the European Union (EU) (Runge et al., 2022). Policy development for sustainable agriculture in the Netherlands followed a similar path. Upon joining the European Union in 1973, the Netherlands adjusted its agricultural policies to align with the Common Agricultural Policy (CAP). To restore the declining multifunctional landscapes, the country implemented multiple policies over the years, starting with the “Relatienota” in 1975 (Compendium voor de Leefomgeving, 2022). This policy aimed to convert designated agricultural lands into nature conservation areas and introduced payments for management practices on farms to enhance nature. In the late 1980s and early 1990s, environmental concerns led to the promotion of organic farming and biodiversity protection. CAP reforms in the mid-1990s influenced Dutch agricultural policy by emphasizing land-use planning for conservation. Concurrently, technological advancements improved productivity but raised sustainability concerns. Since the early 2000s, agricultural collectives have been mobilized for nature management initiatives in the Netherlands, here farmers address environmental concerns collectively. Moreover, in recent years, the Dutch government has increasingly focused on precision agriculture and digitalization, incentivizing farmers to adopt innovative technologies to optimize resource use, improve productivity, and reduce environmental impact in line with broader sustainability goals.

Currently, many farmers maintain contracts with governments or private parties, aiming to secure and fund the delivery of PGs through sustainable management practices (Harmanny et al., 2025). Despite these efforts, landscape quality continues to face significant challenges (Nationaal Dashboard Biodiversiteit, 2024) as these measures appear to fall short of achieving the desired results, and uncertainty persists regarding the efficacy of management practices in facilitating the delivery of PGs (Tyllianakis and Martin-Ortega, 2021). For instance, a significant decline in farmland bird populations is reported. Despite the implementation of agricultural nature management strategies aimed at supporting farmland breeding birds, there has been a decrease of 45 percent within this group (CBS, 2024). Another example is a farm-scale experiment in southern England assessing the impact of four management practices on six PGs that found no clear effects on biodiversity, water quality, and greenhouse gas emissions (Bullock et al., 2021a). Furthermore, many commonly used farm management practices hinder the delivery of PGs. Examples include the widely acknowledged impact of intensive agricultural practices on biodiversity (Rigal et al., 2023) and health (de la Riva et al., 2023), such as the adverse effect of tillage, pesticide and fertilizer use on soil health and water quality (Blanco-Canqui and Ruis, 2018; Pape Møller et al., 2021).

## Objective and Research Questions

Several studies have explored the effectiveness of sustainable farm management practices applying a range of methodological approaches. Examples include: a Dutch case study evaluating soil management from an economic perspective (Kik et al., 2024); a comparative review of the environmental performances of organic agriculture versus conventional farming (Gomiero et al., 2011); and studies with a specific focus on one management practice (Breeuwer et al., 2009) or PG (Iseyemi et al., 2016). While deliberate implementation of these practices is theoretically supported (Teng et al., 2024; USGS, 2024), their actual effectiveness can be contingent on factors like context, implementation, and goals (Van Ruymbeke et al., 2023). Moreover, these practices may have broader impacts on PGs, either positively or negatively. Hence, it is crucial to evaluate the practical experiences when applied, to assess their overall success in achieving desired outcomes. Prior research indicates that many practices are implemented without a comprehensive consideration of their actual (adverse) effects on PGs (Jayaraman et al., 2021; Maderik et al., 2006). Often, a predefined goal, such as targeting a specific PG, is set for accomplishment by implementing a particular practice. However, literature suggests multiple effects could be assumed (Baaken, 2022), and farmers might perceive unexpected positive or negative spill-over effects (Van Ruymbeke et al., 2023). In other cases, practices are sometimes implemented merely because they theoretically work. For example, Cozim-Melges et al. (2024) show that many agricultural practices are implemented based on theoretical assumptions about their ecological benefits, while in practice they can produce mixed, neutral or even negative effects, meaning that their actual implications for PGs often vary.

Our objective is to expand the knowledge and understanding of effects of different management practices on PG delivery. Both when implemented individually and in combination with other practices. This is achieved by identifying the perceived effects of agri-environmental management practices currently implemented by farmers in the Netherlands. Based on a questionnaire with 163 land managers from the Netherlands, we first explore which management practices are used to contribute to the delivery of targeted PGs. Next, we analyze the perceived spillover effects of these management practices, their effectiveness in boosting the delivery of PGs, and impacts on other aspects of the farm, including e.g., income, yield and amount of labor.

## Methods

### Study Area

#### Agriculture in the Netherlands in numbers

**Fig. 1:**
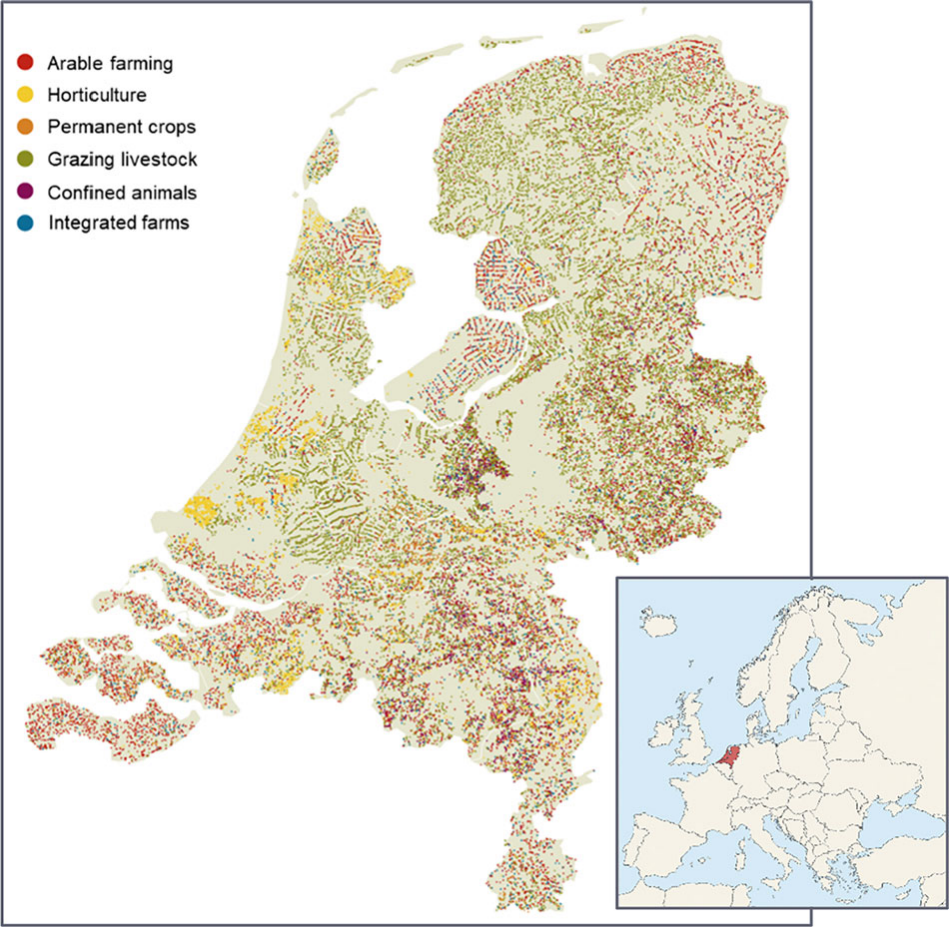
Map of the Netherlands, depicting the spread of the main agricultural specializations (data from Compendium voor de Leefomgeving, 2023) inset shows the location of the Netherlands in red within Europe

Although a small country in terms of surface area, the Netherlands holds the second position globally (after the United States) in agricultural product exports, boasting a total value of 122.3 billion euros annually (Jukema et al., 2023). Over half of the country’s land (54%) is dedicated to agricultural activities (CBS, 2025), significantly impacting the Dutch landscape. Despite a declining number of farms, there is an observed increase in land use per farm due to scale enlargement (CBS, 2023). This study focuses on land-based agriculture in the Netherlands, defined as farms whose production relies entirely or predominantly on the land’s production capacity in close proximity to the farm (Commissie Grondgebondenheid, 2018). The total number of farms in the Netherlands is 50.635 (see Fig. 1) This encompasses 15.251 dairy farms and 11.430 arable farms including flower bulb growers as well as 1240 fruit and tree growers and 60 vineyards (CBS, 2023). Excluded are greenhouses, livestock farming for meat production and poultry farms. Most beef cattle in the Netherlands originates from the dairy industry and is raised in indoor feedlots, making them non-land-based. Consequently, all livestock farming for meat production is excluded from the analysis.

#### Relevant policies

The Dutch agricultural sector has been at the center of numerous discussions, driven by increasing attention to the environmental and societal impacts of intensive farming. An example of this is the key challenge of high level nitrogen emissions from intensive agricultural production, which results in substantial ammonia and nutrient losses that negatively affect biodiversity, air quality, and water systems (de Vries et al., 2023). Policymakers, non-governmental organizations (NGOs), private companies, and consumers, recognize the significance of multifunctionality in the agricultural sector and payment for the provision of PGs (Daniel and Perraud, 2009; OECD, 2015; Viaggi et al., 2021). New policies aim to reduce the environmental footprint while fostering positive contributions. Influential policies include the European Commission’s farm to fork strategy (European Commission, 2020) and similarly, the Dutch government employs specific payments or subsidies to encourage sustainable agricultural practices (Ministerie van LNV, 2018; Rijksoverheid, 2019). The Dutch National Strategic Plan has over 500 million euros earmarked annually to support farmers in agri-environmental measures (Dutch Government, 2023). The European Commission as well as the Dutch government and Dutch private parties are increasingly seeking opportunities for contracts that arrange long-term delivery of PGs, for example, through value chains or land tenure contracts or payment based on results instead of effort.

### Questionnaire

A scoping exercise was done to identify PGs and associated management practices relevant in the Netherlands, resulting in the identification of seven PGs and eleven farm management practices (Table 1). Based on this scoping, a detailed online questionnaire was developed in online survey software Qualtrics and distributed to farmers by e-mail to gather data on their implementation of these practices. The responses were then subjected to analysis to identify patterns and assess the perceived effectiveness of different management practices in supporting the delivery of the identified PGs.

**Table 1:** Definition of agricultural management practices included in the study

Management practice	Definition
Sowing flower strips	Sowing strips with (endemic) wild flowers along the agricultural plots
Nest protection	Marking and protecting the nest of meadow birds to protect them from agricultural activities such as mowing
Sowing herb-rich grassland	Sowing herb-rich grassland instead of mono-crop high-productivity grass
Extended mowing	Extend mowing practices until after the breeding season of meadow birds
Reduce the use of pesticide and fertilizer	Reducing the amount or frequency of fertilizer and pesticides
Reduce tillage	Reduced or no tillage
Grazing of cows	Cows grazing outdoors with access to natural forage, exercise, and ability to express natural behaviors.
Trees and wooded hedge rows	Implement or maintain woodlands, hedge rows or trees
Flooded fields	Inundation of agricultural land during spring to provide habitat for meadow birds
Maintain or restore cultural heritage and facilitate recreation	Maintain or restore cultural heritage and facilitate recreation e.g., by facilitating hiking paths, providing an attractive landscape or maintaining historical features
Maintenance of waterways	Cleaning of waterways and ditches and reducing run off by implementing buffer strips
Other management practices	Other management practices

#### Public goods

While existing classifications of PGs recognize 14–17 PGs (Dwyer et al., 2015; Novo et al., 2017; Pérez‐Soba et al., 2016), this study focuses on seven specific agricultural PGs relevant to Dutch agriculture: 1) climate regulation, carbon storage and air quality 2) soil functionality and protection 3) water quality and availability 4) flood protection and resilience to floods 5) biodiversity 6) farm animal welfare 7) agricultural landscapes and recreation. The elaborate definitions are described in Supplementary Appendix I. Each selected PG is supported by one or more management practices (Section “Identifying management practices”). Other PGs are excluded from this study either because they are generated by land management practices not used in the Netherlands (e.g., Mediterranean olive groves or vineyard contouring and terracing) or because there is limited demand for them such as the provision of wild medicinal plants.

#### Identifying management practices

We inventoried management practices that support the delivery of PGs, for evaluation to the farmers in the questionnaire. In this study, we saw that, traditionally, the focus of Dutch agricultural nature management lies on biodiversity, specifically protecting meadow birds (Table 1). We then hypothesized the effects of the selected management practices on the PGs based on existing literature and reports. See Supplementary Appendix II for an overview of the hypothesized effects. By effects we mean: the perceived changes in the provision or state of PGs resulting from the implementation of agri-environmental management practices. Note that the literature review to support the hypothesis on effects was not exhaustive or systematic, as conducting a comprehensive review was not the focus of this study. It was primarily intended to provide an idea of the goals behind the practices. The management practices included in the study are based on data gathered on practices often implemented as was identified in the systematic web-based search by Harmanny et al. (2025) and is further informed by two key Dutch policy documents: the *Nieuw stelsel agrarisch natuurbeheer* (Melman et al. 2014) and the *Index Natuur en Landschap* (BIJ12 2025), which outline the framework, and recommended farm management practices for Dutch farmland aimed at supporting biodiversity and delivering agri-environmental objectives.

#### Questionnaire development and data collection

The research was embedded in a larger questionnaire on contract solutions for the delivery of PGs, that aimed to form a perspective on acceptability of innovative contract solutions for the delivery of PGs and was completed by1866 farmers in 10 European countries. The full questionnaire contains two parts. The first part aimed to retrieve information on the farmers (e.g., age, gender, education) and the farm (e.g., organic, specialization, amount of workers, succession). The second part focusses on the farmers experience with the implementation of environmental practices on their farm, their attitude towards contract design, and the effects of management practices on PG delivery. A detailed description of the questionnaire can be found in the paper of D’Alberto (2024). Ethics approval was waived by the local ethics committee of Vrije Universiteit Amsterdam.

The questions probing implementation of management practices and farmer’s observation of effects was designed as follows: the respondent was presented with a list of all relevant management practices (Table 1). Here they could indicate which ones they implemented and optionally list the observed effects on the selected PGs. Before the respondent was presented with this question, they had already received an explanation on the main concept of PGs, contracts and management practices. The survey question was in Dutch, translated here to English: *‘Different management practices have the potential to contribute to different PGs either positive or negative. Sometimes farmers observe multiple effects on multiple PGs at once. We would like to know which effects you have observed. Please select all the practices you implemented in the past five years, under contract or on your own initiative and additionally describe the effects of the practices you observed (optional).’* Native speakers tested the survey both in English and Dutch. The questions on management practices were only included in the Dutch questionnaire. The link to the questionnaire was distributed to almost 14 thousand Dutch farmers through an agricultural market research agency. The farmers were contacted individually via e-mail, describing the research and inviting them to participate via a link. A reminder e-mail was sent two weeks later. We do not have access or insight into the agency’s database, but were assured that the sample of farmers is representative both spatially and in terms of specialization.

### Data Analysis

The larger questionnaire yielded 264 initial responses a response rate of 2% Of these initial 264 respondents, those who did not answer the complete question on management practices and effects were excluded from the analysis, bringing the total sample of this study to 163, a response rate of 1.2%.

Next, the qualitative answers about management practices and observed effects were coded. In total, there were 11 management practices that could potentially influence seven PGs, resulting in a total of 77 potential effect options. We used a multistep inductive coding approach. After coding all qualitative responses, indicating their impact on each PG as positive, negative, or neutral, we established categories for alternative responses. These encompassed situations where there were no effects, the practice proved ineffective, uncertainty in assessment, does not apply (e.g., grazing of cows for arable farmers), adherence to customary or obligatory norms (e.g., waterway management mandated by water boards), or instances where participants did not provide information on effects. Additionally, we introduced a separate category for any additional effects noted by respondents not specifically related to PGs. These supplementary effects included general observations, variations in costs, fluctuations in yields (lower, maintained, or higher), crop damage, changes in labor requirements, and alterations in pesticide and/or fertilizer usage. We then included a general assessment of effects, such as *‘works well*’. To increase reliability of the coding we asked three experts in the field to code a random sample with our coding sheet and compared this to our answers. There were no differences in coding found in these random samples. We then calculated descriptive statistics for the respondents on gender, age, education, farm specialization, future perspective and agricultural subsidies. Subsequently, we analyzed adoption and co-occurrence by counting the co-occurrence of management practices.

## Results

### Descriptive Statistics Respondents

The 163 valid survey respondents had diverse demographic and agricultural backgrounds (Table 2). In terms of gender, the sample predominantly consists of males (90%), while females constitute 9%, and 1% chose not to answer. Historically, farming has been male-dominated, although there has been a gradual increase in the number of female farmers in the Netherlands, which now amounts to 28% (CBS, 2018). The respondents are mostly aged between 40 and 60, while the age distribution of farmers often skews towards older demographics. Many farmers are older than 40, with a significant portion falling into the 50–60 age range. Regarding agricultural specialization, the sample exhibits a wide range of expertise, with dairy farmers being the most prevalent with 57% (compared to 30% in the Netherlands), a quarter has an arable farm (actual share is 23%) and the remaining respondents have various specializations such as fruit and horticulture (Table 2). Participants overwhelmingly expressed a long-term commitment to farming, with 67% anticipating continuation for more than 10 years. Concerning succession, 92% of participants provided filtered responses, because this question was only asked when it was indicated that activity continuation would be less than 5 years. Regarding agricultural subsidies or payments, a significant portion of the sample (71%) reports receiving direct payments, while 14% receives subsidies through rural development plans, and 15% reports no payments.

**Table 2:** respondent characteristics (*N* = 163)

Variable	Options	Value
Gender	Other	0%
	Male	90%
	Female	9%
	No answer	1%
Age	21–40 years	15%
	41–60 years	55%
	>61	18%
	No answer	12%
Education	Primary	1%
	Upper secondary	7%
	Post secondary	53%
	Bachelor or equivalent	31%
	Master’s or equivalent	6%
	Doctoral equivalent	1%
Specialization	Specialist cereals, oilseeds and protein crops	1%
	General field cropping	25%
	Horticulture	9%
	Fruit	2%
	Various permanent crops combined	1%
	Dairy	57%
	Mixed farming	4%
	Other	1%
Future perspective: expected continuation of farming activity	Between 1 and 5 years	8%
	Between 5 and 10 years	15%
	More than 10 years	67%
	Do not know/never thought about it	10%
Future perspective: successor	Yes in my family	2%
	Yes, not in my family	1%
	No, not been able to find a successor	4%
	Not decided yet/never thought about it	1%
	Filtered, continuation more than five years	92%
Agricultural subsidies/payment (multiple answers possible)	Yes, direct payment	71%
	Yes, rural development plan	14%
	No payments received	15%

### Descriptive Statistics Management Practices

**Fig. 2:**
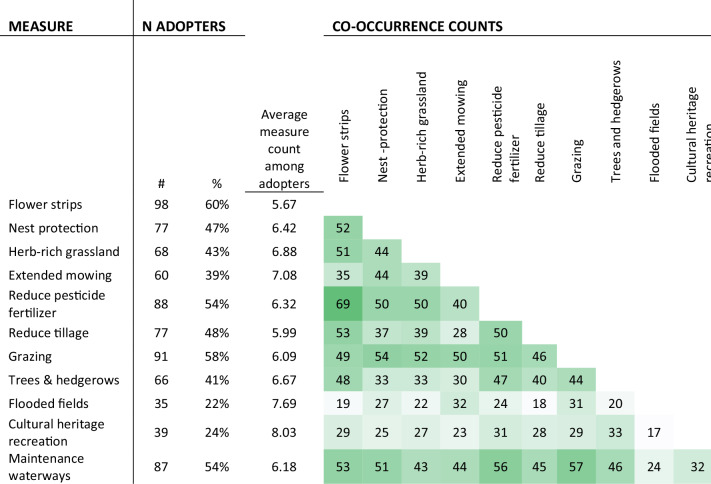
Statistics on the adoption of measures and their co-occurrence (i.e., how many and which measures were implemented together). *Measure* refers to implemented agri-environmental management practices. *N adopters* indicates the number of farmers implementing a measure, reported in absolute count (#) and percentage (%), followed by the *average measure count*. Green shading indicates the degree of co-occurrence, with darker shades representing higher levels of co-occurrence

A diverse range of implemented management practices is reported by respondents, with half implementing 3–6 practices and 10 farmers incorporating all 11 practices. Flower strips emerge as the most widely adopted measure, implemented by 98 respondents, followed closely by grazing (91 respondents), reduced fertilizer and pesticides (88), and waterway maintenance (87). Conversely, less commonly implemented measures are field flooding (35 respondents) and the preservation of cultural heritage or facilitating recreation (39 respondents). Respondents implementing these less common measures tend to have a higher overall number of practices in place compared to those without (Fig. 2). Additionally, respondents with only one management practice often choose flower strips (4), nest protection (1), reduced tillage (5), grazing (3), or waterway maintenance (2). Among those with precisely two practices (17), half combines flower strips, nest protection, reduced tillage, grazing, or waterway maintenance, with the remaining half combining two of the aforementioned single practices.

Common combinations of management practices can be seen among respondents, showcasing the synergy of sustainable agricultural approaches. Noteworthy pairings include the integration of reduced pesticides and fertilizer use with flower strips (*n* = 69), waterway maintenance coupled with grazing (*n* = 57), and the combination of waterway maintenance and reduced pesticides (*n* = 56). Other prevalent combinations are grazing with nest protection (*n* = 54), flower strips with reduced tillage or waterway maintenance (*n* = 53), and a comprehensive approach involving flower strips, nest protection, grazing, and herb-rich grasslands (*n* = 52). Exploring the less common measures, flooded fields are often complemented by extended mowing and grazing, while cultural heritage practices are frequently implemented alongside trees and hedgerows or reduced pesticide strategies. Respondents with more than two practices display a diverse array of combinations, highlighting the adaptability and variety of sustainable agricultural management strategies within the sample.

### Perceived Effects on Public Goods

**Fig. 3:**
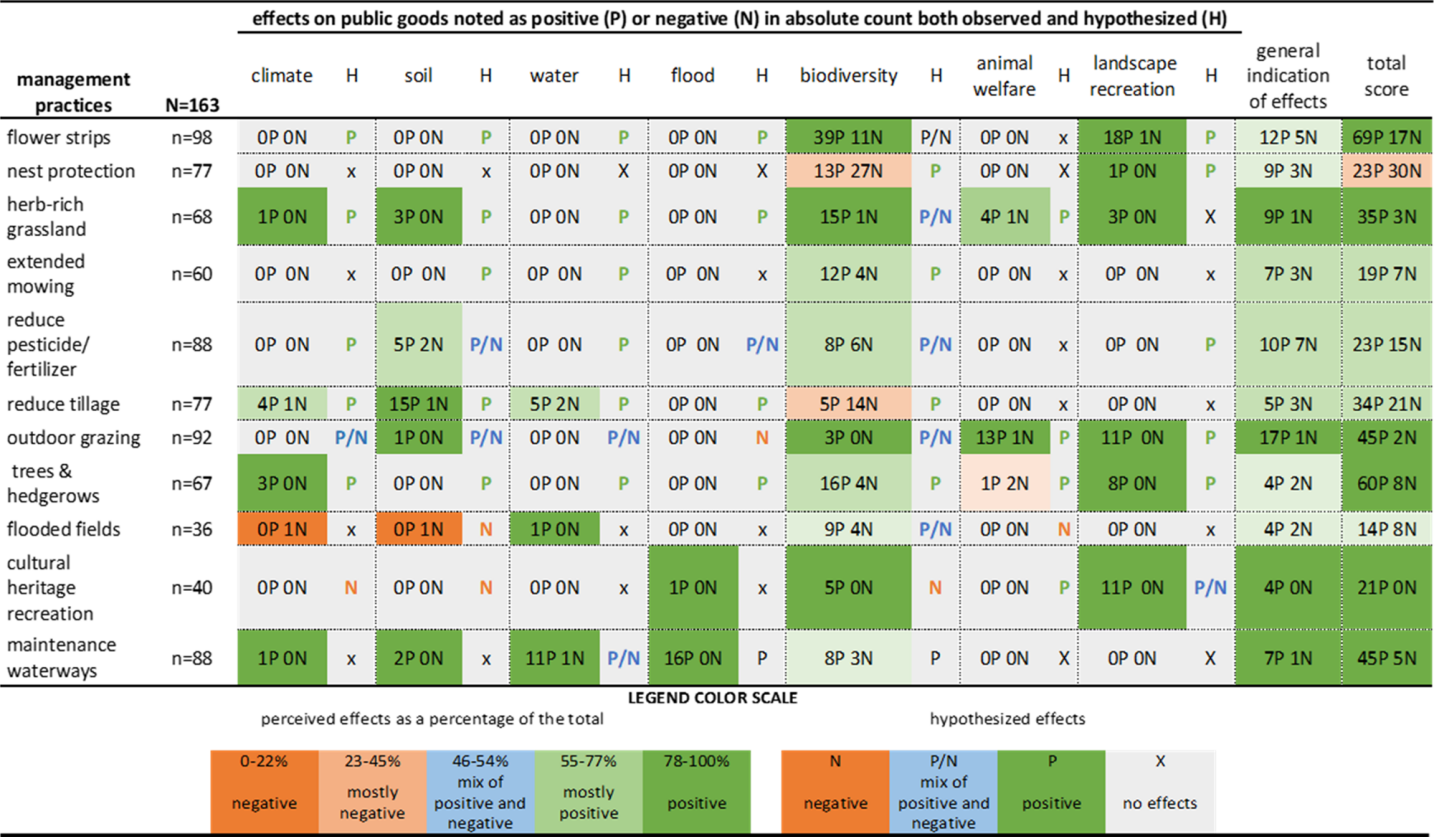
Effects of management practices on public goods, reported by the respondents (*N* = 163). For example, 5P 2N refers to 5 positive and 2 negative effects on a public good. Next to the reported effects are the hypothesized effects *(H)* for comparison. *General indication of effects* refers to the non-specific reports of effects (e.g. *''works great''*). The last column *total score* combines the number of all effects that were noted for a management practice

When asking participants to select which of the 11 agricultural management practices they had implemented on their farm and (optionally) provide details about the observed effects of these practices on seven PGs, respondents indicated no effect for 41 of the 77 combinations of management practice and effect on PGs (Fig. 3). This outcome was anticipated in certain instances. For example, the lack of noted effects for nest protection on water quality or flood protection was consistent with our expectations as it is almost inherent to the practice to have no effect on these PGs. However, in other cases, where positive effects on water quality were anticipated with reduced pesticide and fertilizer use, reported effects were absent.

Additionally, the breakdown of effect options of reduced pesticide and/or fertilizer use on the different PGs revealed positive outcomes 23 times and negative outcomes 15 times. Positive effects of flower strips were reported most frequently, in 69 cases. Among these, 39 respondents reported positive effects on biodiversity, 18 on landscape recreation, and 12 respondents reported positive effects in general. Trees and hedgerows (60 positive), grazing, and waterway maintenance (both with 45 positive effects) were also often perceived positive by the respondents.

While there are general similarities between the effects reported by farmers and those found in the literature, discrepancies also emerge. An example is the effect of no- or reduced tillage where survey respondents report predominantly negative impacts, specifically on biodiversity, despite literature suggesting potential positive effects on soil structure, water retention and reduces erosion. Next, for cultural heritage and recreation, literature indicated a negative impact on biodiversity, but respondents reported a positive effect, challenging the anticipated disruption of local flora and fauna by increased human activity. Contrary to this, many of the 77 observed effect of our survey respondents are in line with the hypotheses, including e.g., for grazing of cows, hypothesized to positively influence biodiversity, animal welfare, and landscape and recreation (Fig. 3).

### Benefits and Trade-offs

The practice of sowing flower strips is implemented most frequently (*n* = 98) showing the most frequent positive effects for enhancing biodiversity, landscape and recreation. Although positive effects on other PGs, like soil health and water quality through buffer zones were expected, these are not reported. Respondents often note that they observed increased activity of insects and other animals and that they receive positive remarks by passersby; this was also found by Bullock et al. (2021b). In contrast, nest protection is characterized by the highest number of listed negative effects (27 times), accompanied by 18 respondents stating that the practice proved entirely ineffective. A clear trend emerges in farmers’ qualitative responses, highlighting concerns such as making it easier for predators to locate nests and questioning the effectiveness without allowing to hunt predators like foxes. In addition, it was mentioned that some birds were ‘’saved” because of the nest protection but did not grow into maturity because of too limited resources. Despite literature suggesting positive effects on biodiversity, practical experiences indicate otherwise.

Reduced tillage is associated with positive or mostly positive effects on climate, soil, and water (Blanco-Canqui and Ruis, 2018), but a predominantly negative impact on biodiversity is noted. Farmers express that while reduced tillage may benefit soil health, it simultaneously increases weed pressure and pests, disrupting the biodiversity balance. Some farmers mention decreased yields and reduced soil permeability in the short term, creating challenging soil management conditions (see Supplementary Appendix III for an overview of other effects noted by respondents beyond those related to public goods). Reduced pesticide and fertilizer usage are reported to have a predominantly positive effect on soil and biodiversity (Geiger et al., 2010; Pape Møller et al., 2021). However, many farmers report lower yields as a consequence, along with increased costs. Based on the literature we can assume spillover effects such as minimizing runoff and leaching of harmful chemicals into water bodies, but this was not explicitly reported and therefore not coded as such.

Trees and hedgerows are reported to have a positive effect on climate, biodiversity, and landscape and recreation. However, it was also reported to require more labor and negatively impacting farm animals. Cows seeking shade under trees often face muddy and wet conditions, increasing the risk of inflammation of udders and hooves. Maintenance of waterways has only positive effects and 2 instances of no effects, while grazing is reported with only positive effects and three instances of no effects. Many farmers note the water board requires waterway maintenance. These practices positively influence water quality and flood protection and also contribute to biodiversity. Flooded fields appear to exert a mostly positive influence on biodiversity, especially for meadow birds. However, prolonged inundation was reported to negatively impact soil quality in line with the hypothesized effect.

## Discussion

### Interpretation of Results

This study aimed to assess the impact of a range of agricultural management practices on seven PGs. We sought to determine the farmers’ perceptions of effects and if they align with the hypothesis, revealing both congruities and disparities. Farmers’ observation of effects varied, encompassing negative, positive, or a combination of both. All the listed management practices are actively employed by farmers to facilitate PG delivery, with adoption rates ranging from 60% (flower strips) to 20% (inundation of fields). Overall, the main focus of the implemented measures is biodiversity enhancement. This focus is driven by policy and the value chain, particularly within the dairy sector as the prevalence of certain practices, such as grazing for dairy farmers, is attributed to robust support from value chain contracts and Dutch eco schemes (Jongeneel and Gonzalez-Martinez, 2023). Additionally, waterway maintenance often results from mandatory directives imposed by water boards. Reduced fertilizer and pesticide use may be linked to derogation abandonment or political pressure. Flower strips, integral to Common Agricultural Policy measures (Schmidt et al., 2022), receive substantial support for arable land within Dutch eco schemes (Jongeneel and Gonzalez-Martinez, 2023). The implementation of measures appears contingent upon their invasiveness, with easily adoptable practices having a lower threshold compared to more complex ones (Lastra-Bravo et al., 2015). For instance, sowing flower strips is a relatively easy measure to implement, while activities like extended mowing entail larger trade-offs with production, and inundation of fields poses challenges that require coordination with water boards, neighbors, and significant investments in equipment such as pumps.

We hypothesized that the effects of management practices extend beyond the PGs they target. Our findings reveal not only positive spillover effects but also negative ones. Beyond influencing various PGs, respondents reported additional impacts on yield, labor requirements, and costs, underscoring the extensive reach of these measures. Specifically, reductions in pesticide and/or fertilizer use were found to adversely affect yield and increase costs. Extended mowing, aimed at safeguarding meadow birds, showed the least impact according to respondents. The context-specific nature of these management practices suggests that comprehensive effectiveness often requires multiple measures rather than a single approach. In addition to contributing insights on the effectiveness of specific agri-environmental measures, our study provides on-the-ground understanding of why certain measures may fail. For instance, nest protection intended for meadow birds (targeting the PG biodiversity) was reported to have unintended negative consequences, as farmers observed that marking nests made them more vulnerable to predators like foxes. Another notable measure, the planting of flower strips alongside fields, was praised for enhancing biodiversity, particularly insect populations, and improving the aesthetic appeal of landscapes (PG cultural heritage and recreation). However, it also led to increased weed pressure and greater pesticide usage, highlighting the complex trade-offs involved. A more holistic and diverse field planning approach could mitigate these challenges, emphasizing the need for careful considerations.

### Synergies Between Practices

Farmers who embrace more invasive measures often implement multiple measures over time. This aligns with the concept of farmers “accumulating” measures, wherein they initiate with one measure and, upon witnessing positive outcomes, integrate additional practices. This is referred to as the portfolio effect, as noted by Weltin et al. (2021). Moreover, engaging in a subsidy scheme may serve as a catalyst, potentially lowering the threshold for implementing additional measures while increasing earnings. The strategy of adopting multiple measures is particularly evident in cases where these measures contribute together to the support of the same PG. For instance, combining herb-rich grassland and flooded fields not only enhances meadow bird food provisioning but also provides nest protection, potentially improving nest success. This aligns with the inherent nature of subsidy schemes, which encourages a comprehensive approach to addressing multiple aspects of PG support. Similarly, practices such as sowing flower strips and planting or maintaining trees and hedgerows serve a dual purpose by attracting pest predators, thereby, in theory, reducing the necessity for pesticides. However, in this study, farmers did not report reduced pesticide use as a result of these measures.

An additional challenge in implementing practices lies in the interdependence among neighboring farms, which is especially evident in initiatives targeting water quality improvement. For instance, our findings indicate that individual farmers who reduced their use of fertilizers and pesticides did not observe an improvement in local water quality. This limited impact can largely be attributed to the interconnectedness of waterways, where isolated actions are insufficient to produce substantial improvements. To achieve meaningful progress in water quality across a watershed, it is essential for all farms within the region to adopt similar management practices, as waterborne pollutants readily transfer through shared watercourses. This aligns with previous studies (Mitsch and Day, 2006; Wakwella et al., 2023) on effective water quality improvements, it is crucial to implement interventions at multiple scales and utilize policy instruments that manage collectively. A similar phenomenon was observed with initiatives aimed at protecting meadow bird populations. Although farmers reported an increase in nests and chick survival when adopting measures like habitat conservation, they noted that the juvenile birds faced food shortages as they matured. This was due to a lack of participation in the program by neighboring farms, which restricted the availability of resources across the broader landscape. These findings underscore the importance of coordinated, landscape-scale management practices to enhance the effectiveness of conservation efforts across interconnected farm areas.

In several management practices, we observed a gap between findings reported in the literature and actual outcomes in practice. This is particularly evident in “nest protection” where the intended goal is to enhance breeding success and promote biodiversity within agricultural ecosystems. While a positive effect on both biodiversity and landscape value were expected, our observations reveal different results. One contributing factor may be the lack of coordinated measures; for instance, some respondents noted the need for complementary actions, such as predator control, to enhance the effectiveness of nest protection. Differences in perception may also impact outcomes; for example, Maas et al. (2021) found that scientists and farmers often value information sources differently when making decisions about biodiversity, agri-environmental schemes, and conservation measures. While scientists tend to prioritize research-based data, farmers place greater trust in insights from government and agricultural industry sources. This divergence in information priorities can influence how practices are applied and perceived, potentially impacting their effectiveness on the ground.

### Limitations and Discussion of the Methods

Our survey among farmers builds on approaches applied by studies on payment for ecosystem services (e.g., Rodríguez-Ortega et al., 2018), adoption of agri-environmental management practices (Thompson et al., 2022) and their effects on PGs or ecosystem services delivered by agriculture (e.g., Hološková et al., 2025). Existing approaches often concentrate on individual PGs such as soil functionality (Blanchy et al., 2023), or water quality (Beaudoin et al., 2021), but offer limited insights into the diversity of management practices and their effects across multiple PGs. Our study adds to this by providing detailed information on the perceived effects of different management practices on a range of PGs within a distinct region. and offers valuable insights into the perceived effectiveness of management practices. However, several limitations should be considered. Firstly, sampling bias may affect the study’s generalizability, with certain groups or characteristics being either overrepresented or underrepresented. For example, dairy farmers are overrepresented in our sample while other specializations are underrepresented (CBS, 2018). Additionally, the response rate of the survey was relatively low. The questionnaire yielded 264 initial responses, a response rate of 2% and after excluding those who did not answer the open questions on perceived effects, 163 responses remained (1.2%). Open-ended questions, while useful for capturing a broad range of perspectives and avoiding steering respondents, often require more effort to answer and can therefore reduce overall response rates. Low participation is common in online surveys among farmers. Previous research by Zahl-Thanem et al. (2021) shows that email surveys among farmers typically yield lower response rates than traditional postal surveys. However, comparing the entire survey revealed very few significant differences between the two samples. Similarly, a study examining agricultural innovation adoption reported a final sample of *N* = 196 for an online survey among Dutch and German farmers (Tensi et al., 2022), illustrating that relatively low response rates are not uncommon in voluntary farmer surveys. Survey participation may further be influenced by factors such as timing within the agricultural production cycle, survey length, perceived relevance of the topic, and trust in the organization conducting the research (Pennings et al., 2002). The possibility of non-response bias should be considered when interpreting the results, as it hampers our ability to detect subgroup differences. Lastly, although respondents were explicitly asked to independently describe the effects of each measure, the study does not ascertain the extent to which interactions or synergies between practices may have influenced the results.

Despite these limitations, a clear pattern emerges in the responses provided by farmers, particularly in the qualitative answers. This is illustrated by the negative impacts concerning nest protection, where nearly all respondents state the presence of predators as a primary constraint on this measure. This underlines the challenge inherent to policy implementations for management practices: their effectiveness can be influenced by various factors, including context, implementation strategies, goals and other practices. Many practices may seem promising on paper but encounter practical obstacles due to contextual factors. While these management practices theoretically should yield positive outcomes, farmers observe their inefficacy in contexts already marked by degradation (e.g., nitrogen issues and declining biodiversity on a broader scale). This emphasizes the necessity for transformative change and closing the gap between scientific knowledge and its practical implementation.

### Implications and Conclusions

Our study contributes by empirically evaluating farmers’ perceptions of a broad range of agricultural management practices and their effects on multiple PGs, an area that has received limited structured attention in previous survey research. The evaluated expected effects provide a foundation for future studies, e.g., to replicate similar evaluations on a larger scale. Information about impacts of management practices as observed in the field can inform the design of agricultural policies, subsidy schemes, and conservation programs. While the sample size is limited and results should be interpreted with caution, the findings offer a starting point for research linking perceptions to actual ecological outcomes and examining trade-offs and synergies of management practices among multiple PGs. By highlighting the gap between practices deemed effective in the literature and farmers’ perceptions, the study emphasizes the need for integrated behavioral, ecological, and policy-focused research to support evidence-informed agricultural management and PG delivery.

The outcomes of different agricultural management practices reveal the importance of addressing both perception and behavioral factors. Farmers and scientists often prioritize different sources of information and expectations for outcomes, which can influence the effectiveness and adoption of practices. Improved communication and shared understanding, such as through workshops or co-developed strategies, could enhance alignment and uptake of biodiversity-focused practices and provide farmers with broader information on the effect of the measures than the anecdotal evidence that is often underlying perceptions. These findings also highlight important considerations for the financing and design of PG delivery particularly in light of recent moves toward results-based contracts (where a farmer receives payment for results instead of input) and private sector involvement in PG funding. Subsidy designs also play a critical role, as seen in the success of bundled “package” approaches, where complementary measures (like flower strips paired with meadow bird conservation) create synergistic effects and gain greater acceptance. Moving towards payment structures that incentivize packages rather than individual measures could reinforce these synergies and help generate more substantial, landscape-scale benefits. The effectiveness of measures can have both positive and negative spillover effects, underlining the need for policies that account for both co-benefits and potential conflicts. For example, while some practices, like flower strips, yield widely reported ecological gains, others may have mixed or indirect impacts. Payment schemes should consider these complexities and set realistic, flexible goals that acknowledge both direct and secondary effects of management practices. Additionally, as companies show growing interest in subsidizing PGs, structured financial incentives could support sustainable agriculture in ways that align with environmental objectives, fostering a more resilient agricultural landscape.

## Supplementary information

Appendix I

Appendix II

Appendix III

## Data Availability

The surveys that support the findings of this study are available, but restrictions apply to the availability of these data, which were used under licence for the current study and so are not publicly available. The data are, however, available upon request.
