# Supplementary material for: Perceived Effects of Agri-Environmental Management Practices on Public Good Delivery
Source: Environ Manage. 2026 Jun 29;76(7):234. doi: 10.1007/s00267-026-02485-2 (PMC13314725; doi:10.1007/s00267-026-02485-2)
Supplement: Supplementary file 1 — Appendix I [file 267_2026_2485_MOESM1_ESM.docx]

# Appendix I definition and classification of public goods

| **PUBLIC GOOD** |  | **ENVIRONMENTALLY AND SOCIALLY BENEFICIAL OUTCOMES** |
| --- | --- | --- |
|  |  |  |
| Climate regulation \| Carbon storage \| Air quality |  | Achieving or maintaining minimized levels of harmful emissions and odor levels and maximize carbon sequestration/storage. |
|  |  |  |
| Soil functionality and protection |  | Achieving or maintaining good functionality through good biological and geochemical conditions of soil and minimize soil degradation and erosion. |
|  |  |  |
| Water quality and availability |  | Achieving or maintaining good ecological status of surface water and good chemical status of groundwater. In addition to regular supply of water i.e. avoidance of water scarcity. |
|  |  |  |
| Flood protection and resilience to floods |  | Achieving or maintaining minimization of impacts of potential floods (tightly linked to water availability through the management of water flows). |
|  |  |  |
| Biodiversity |  | Achieving or maintaining the presence of diverse and sufficiently plentiful species and habitats (ecological diversity) and farmland biodiversity/agrobiodiversity in addition to megafauna habitat and shelter for game; high levels of pollination; high levels of biological pest and disease prevention and minimization of the impacts of potential outbreaks using biodiversity. |
|  |  |  |
| Farm animal welfare |  | Farm animal welfare: achieving or maintain the implementation of high farm animal welfare practices. |
|  |  |  |
| Agricultural landscapes and recreation |  | Landscape character and cultural heritage: maintaining or restoring a high level of landscape character and cultural heritage. Additionally achieve or maintain possibilities for outdoor recreation. |
