# Supplementary material for: Perceived Effects of Agri-Environmental Management Practices on Public Good Delivery
Source: Environ Manage. 2026 Jun 29;76(7):234. doi: 10.1007/s00267-026-02485-2 (PMC13314725; doi:10.1007/s00267-026-02485-2)
Supplement: Supplementary file 2 — Appendix II [file 267_2026_2485_MOESM2_ESM.docx]

# Appendix II Expected effects of agri-environmental management practices on public goods

| **MANAGEMENT PRACTICE** | **EXPECTED EFFECTS ON PUBLIC GOODS** | | | | | | |
| --- | --- | --- | --- | --- | --- | --- | --- |
|  | **Climate** | **Soil** | **Water** | **Flood** | **Biodiversity** | **Animal welfare** | **Landscapes & recreation** |
| **Sowing flower strips**  *with (endemic) species along agricultural plots* | + climate regulation by diverse plant species contribute to carbon sequestration | + reducing erosion; enhancing water retention; more microbial activity | + buffer zones filtering runoff; preventing soil erosion | + reducing surface runoff; enhancing soil permeability | + habitat and food sources pollinators/wildlife  - Wrong plant choice invasive species potentially lead to biodiversity loss |  | + improves visual appeal of agricultural landscapes; attracting visitors |
| **Sowing herb-rich grassland** *instead of mono-crop high productivity grass* | + climate regulation by diverse plant species contribute to carbon sequestration | + reducing erosion; enhancing water retention; more microbial activity | + buffer zones filtering runoff; preventing soil erosion | + reducing surface runoff; enhancing soil permeability | + providing habitat and food sources  - wrong plant choice invasive species potentially lead to biodiversity loss | + diverse forage options and a more natural environment |  |
| **Nest protection** *marking and protecting the nest of meadow birds to protect them from agricultural activities* |  |  |  |  | + nest protection safeguards the reproductive success of bird species |  | + attracting birdwatchers |
| **Extended mowing** *practices until after the breeding season of meadow birds* |  | + reducing soil disturbance and supporting plant growth | + promoting vegetation along water bodies |  | + providing habitat and food sources for various species |  |  |
| **MANAGEMENT PRACTICE** | **EXPECTED EFFECTS ON PUBLIC GOODS** | | | | | | |
| **Flooded fields** *inundation of agricultural land during spring to provide habitat for meadow birds* |  | - Prolonged inundation may lead to oxygen depletion in the soil, negatively impacting soil health |  |  | + providing habitat for aquatic and wetland species  - Intense or prolonged inundation may disrupt nesting and foraging habitats, impacting certain species negatively | - prolonged inundation may limit access to grazing and disrupt farm activities, negatively impacting animal welfare. |  |
| **Reduction pesticide and/or fertilizer** *reducing the amount or frequency* | + minimizing greenhouse gas emissions | + improve soil functionality by enabling natural nutrient cycling, microbial diversity, and reducing the risk of soil contamination  - reduced fertilizer use may lead to nutrient deficiencies in crops, affecting soil fertility. | + minimizing runoff and leaching of harmful chemicals into water bodies. | + minimizing nutrient runoff and soil erosion  - if pest-related crop damage increases, it might indirectly impact soil stability and contribute to runoff during heavy rainfall. | + preserving natural predators and minimizing harm to non-target species  - may lead to increased pest pressure, impacting certain plant and animal species negatively |  | + safer recreational spaces |
| **Tillage** reduced or no tillage | + helps sequester carbon in the soil | + improves soil structure, water retention, and reduces erosion. | + minimizes soil disturbance, reducing sediment and nutrient runoff into water bodies | + Improved soil structure from reduced or no tillage can contribute to better water infiltration and flood protection | + supports biodiversity by preserving soil organisms |  |  |
| **MANAGEMENT PRACTICE** | **EXPECTED EFFECTS ON PUBLIC GOODS** | | | | | | |
| **Maintenance of waterways** *Cleaning of waterways and ditches and reducing run off by implementing buffer strips* |  |  | + enhances water quality by preventing sedimentation  - short-term water quality disturbance during maintenance | + Well-maintained waterways contribute to effective flood protection | + supports diverse aquatic habitats |  |  |
| **Woodwall trees** *Implement or maintain woodwalls, hedge rows or trees* | + sequestering carbon | + stabilize soil, prevent erosion; enhance nutrient cycling | + Vegetative barriers improve water quality by filtering runoff | + help mitigate floods by reducing runoff | + provide habitats for biodiversity  -provides habitat for predators may impact (young) meadow birds | + offers shade and shelter for animals | + enhance landscape aesthetics |
| **Maintain or restore cultural**  **heritage and facilitate recreation** *and facilitate recreation e.g. by facilitating hiking paths, providing an attractive landscape or maintaining historical features* | - increased human activity may contribute to localized environmental impacts | - intensive recreational use may lead to soil compaction and erosion |  |  | - increased human activity may disrupt local flora and fauna | + opportunities for education on animal welfare. | + contribute to landscape aesthetics and provide recreational opportunities.  - Overuse or inadequate management may impact landscape integrity and recreation |
| **Grazing** *enable grazing and natural behavior for livestock* | + healthy grasslands that sequester carbon  - Overgrazing may lead to soil degradation and reduced carbon sequestration | + can enhance soil structure; nutrient cycling  - may lead to soil compaction and erosion | + can contribute to vegetation control along water bodies, improving water quality  - Overgrazing near water sources may lead to sedimentation and nutrient runoff | - may decrease vegetation cover, increasing the risk of soil erosion and impacting flood protection | + can support diverse ecosystems  - Uncontrolled or excessive grazing may negatively impact native plant and animal species | + provides natural foraging opportunities for livestock, contributing to animal welfare | + contribute to the aesthetic appeal and cultural value of rural areas |
