# Supplementary material for: Perceived Effects of Agri-Environmental Management Practices on Public Good Delivery
Source: Environ Manage. 2026 Jun 29;76(7):234. doi: 10.1007/s00267-026-02485-2 (PMC13314725; doi:10.1007/s00267-026-02485-2)
Supplement: Supplementary file 3 — Appendix III [file 267_2026_2485_MOESM3_ESM.docx]

# Appendix III Other perceived effects

Other effects noted by respondents beyond those related to public goods. *NA* indicates no answer.

| **MAMNAGEMENT PRACTICE** | **N=163** | **OTHER EFFECTS NOTED** | | | | | | | | |
| --- | --- | --- | --- | --- | --- | --- | --- | --- | --- | --- |
|  |  | other effect | higher costs | lower costs | maintained or higher yield | lower yield or damaged crops | more labor | less labor | increase pesticide/fertilizer | decrease pesticide/fertilizer |
| flower strips | n=98 | 3 | 1 | NA | 1 | 3 | 1 | NA | 1 | 1 |
| nest protection | n=77 | NA | NA | NA | NA | NA | 2 | NA | NA | NA |
| herb-rich grassland | n=68 | 2 | 2 | NA | 1 | 5 | 2 | NA | NA | NA |
| extended mowing | n=60 | 1 | 2 | NA | NA | 3 | 1 | NA | NA | NA |
| reduce pesticide and/or fertilizer | n=88 | 1 | 5 | 3 | 2 | 11 | 2 | NA | NA | NA |
| reduce tillage | n=77 | NA | NA | 3 | NA | 3 | 1 | NA | 8 | NA |
| grazing | n=92 | NA | 1 | 1 | NA | 4 | 3 | 1 | NA | NA |
| trees & hedgerows | n=67 | 3 | 3 | NA | 1 | 2 | 4 | NA | NA | NA |
| flooded fields | n=36 | 1 | NA | NA | NA | 2 | 1 | NA | NA | NA |
| cultural heritage recreation | n=40 | NA | 3 | NA | NA | NA | 4 | NA | NA | NA |
| maintenance waterways | n=88 | NA | NA | NA | 1 | NA | 1 | 2 | NA | NA |
